# RETRACTION: Effects of Negative Pressure Wound Therapy on Surgical Site Wound Infections after Cardiac Surgery: A Meta‐Analysis

**DOI:** 10.1111/iwj.70320

**Published:** 2025-03-06

## Abstract

**RETRACTION:** TaoY. , ZhangY. , LiuY. , and TangS. , “Effects of Negative Pressure Wound Therapy on Surgical Site Wound Infections after Cardiac Surgery: A Meta‐Analysis,” International Wound Journal 21, no. 2 (2024): e14398, 10.1111/iwj.14398.PMC1082459837740679

The above article, published online on 23 September 2023, in Wiley Online Library (http://onlinelibrary.wiley.com/), has been retracted by agreement between the journal Editor in Chief, Professor Keith Harding; and John Wiley & Sons Ltd. Following an investigation by the publisher, all parties have concluded that this article was accepted solely on the basis of a compromised peer review process. The editors have therefore decided to retract the article. The authors did not respond to our notice regarding the retraction.

**RETRACTION:** Y. Tao , Y. Zhang , Y. Liu , and S. Tang , “Effects of Negative Pressure Wound Therapy on Surgical Site Wound Infections after Cardiac Surgery: A Meta‐Analysis,” International Wound Journal 21, no. 2 (2024): e14398, 10.1111/iwj.14398.PMC1082459837740679

The above article, published online on 23 September 2023, in Wiley Online Library (http://onlinelibrary.wiley.com/), has been retracted by agreement between the journal Editor in Chief, Professor Keith Harding; and John Wiley & Sons Ltd. Following an investigation by the publisher, all parties have concluded that this article was accepted solely on the basis of a compromised peer review process. The editors have therefore decided to retract the article. The authors did not respond to our notice regarding the retraction.

